# Astroglial and oligodendroglial markers in the cuprizone animal model for de- and remyelination

**DOI:** 10.1007/s00418-022-02096-y

**Published:** 2022-04-05

**Authors:** Maria de los Angeles Castillo-Rodriguez, Stefan Gingele, Lara-Jasmin Schröder, Thiemo Möllenkamp, Martin Stangel, Thomas Skripuletz, Viktoria Gudi

**Affiliations:** 1 Department of Neurology, Hannover Medical School, Carl-Neuberg-Str-1, 30625 Hannover, Germany; 2 Center for Systems Neuroscience, Hannover, Germany

**Keywords:** Multiple sclerosis, Demyelination, Astrocytes, Cuprizone

## Abstract

Myelin loss with consecutive axon degeneration and impaired remyelination are the underlying causes of progressive disease in patients with multiple sclerosis. Astrocytes are suggested to play a major role in these processes. The unmasking of distinct astrocyte identities in health and disease would help to understand the pathophysiological mechanisms in which astrocytes are involved. However, the number of specific astrocyte markers is limited. Therefore, we performed immunohistochemical studies and analyzed various markers including GFAP, vimentin, S100B, ALDH1L1, and LCN2 during de- and remyelination using the toxic murine cuprizone animal model. Applying this animal model, we were able to confirm overlapping expression of vimentin and GFAP and highlighted the potential of ALDH1L1 as a pan-astrocytic marker, in agreement with previous data. Only a small population of GFAP-positive astrocytes in the corpus callosum highly up-regulated LCN2 at the peak of demyelination and S100B expression was found in a subset of oligodendroglia as well, thus S100B turned out to have a limited use as a particular astroglial marker. Additionally, numerous GFAP-positive astrocytes in the lateral corpus callosum did not express S100B, further strengthening findings of heterogeneity in the astrocytic population. In conclusion, our results acknowledged that GFAP, vimentin, LCN2, and ALDH1L1 serve as reliable marker to identify activated astrocytes during cuprizone-induced de- and remyelination. Moreover, there were clear regional and temporal differences in protein and mRNA expression levels and patterns of the studied markers, generally between gray and white matter structures.

## Introduction

Multiple sclerosis (MS) is an autoimmune demyelinating disease of the central nervous system (CNS) characterized by a large confluence of plaques of primary demyelination with variable axonal degeneration, inflammation, and astrogliosis (Lumdsen 1970; Lassmann et al. 2012). The subsequent regenerative mechanism to restore the normal myelin structure, known as remyelination, is only partial or fails completely, leading to a chronic progressive form of the disease within 10–15 years (Patrikios et al. 2006; Antel et al. 2012; Lublin et al. 2014). The prominent presence of astrocytes in demyelinating plaques suggests them to be amongst the main effectors in MS pathogenesis (Cambron et al. 2012; Ponath et al. 2018). However, since both protective and detrimental effects have been observed, their exact role remains unclear (Williams et al. 2007; Nair et al. 2008; Moore et al. 2011). Astrocytes provide support and protection for oligodendrocytes and neurons and regulate myelination through the release of a broad range of cytokines, chemokines, and growth factors. Furthermore, astrocytes improve synaptic plasticity, minimize cytotoxicity by glutamate uptake, and insulate brain structures by forming the blood–brain barrier (Kimelberg 2010; Anderson et al. 2016; Kıray et al. 2016). Activated astrocytes in MS lesions are known to secrete a broad range of pro-inflammatory cytokines, in turn attracting immune cells, impeding oligodendrocyte precursor cell (OPC) maturation and forming a physical barrier known as a glial scar that prevents the migration of OPC into the areas of demyelination (Franklin 2002; Sofroniew and Vinters 2010; Correale and Farez 2015).

In certain CNS pathologies, astrocytes acquire an “activated” or “reactive” form, proliferate, and display a prominent hypertrophy of their cellular processes. Immunohistochemical identification of astrocyte activation relies primarily on the upregulation of their cytoskeletal components, the intermediate filaments glial fibrillary acidic protein (GFAP), vimentin (VIM), and nestin (Eliasson et al. 1999; Pekny 2001; Liu et al. 2014). GFAP is the most established marker for astrocytes, even though its expression levels vary considerably between different brain regions and environmental contexts (Eddleston and Mucke 1993; Middeldorp and Hol 2011). This leads to difficulties in the study of different astroglial subpopulations, since astrocytes show broad heterogeneity throughout the CNS. Astrocytes are commonly classified into fibrous white matter and protoplasmic gray matter populations (Oberheim et al. 2012). There are also regional and temporal varieties in the molecular expression patterns of astroglial subpopulations (Nair et al. 2008; Khakh and Sofroniew 2015). Therefore, several astrocytic markers including S100B, aquaporin 4, glutamine synthetase (GS), the aldehyde dehydrogenase 1 family member L1 (ALDH1L1), glutamate/aspartate transporters (GLAST and GLT-1), and connexin 43 have gained importance over recent years (Cahoy et al. 2008; Yang et al. 2011; Verkhratsky and Nedergaard 2018). Lipocalin 2 (LCN2), also known as neutrophil gelatinase-associated lipocalin (NGAL) (Kjeldsen et al. 1993; Cowland and Borregaard 1997), is a small acute-phase protein that can bind and transport hydrophobic molecules and sequester iron (Flower 1996; Flo et al. 2004). It also has effects on cell growth, survival, apoptosis, migration, and differentiation in different tissues including the CNS, and plays a role in cancer progression and angiogenesis (Jang et al. 2013a; Ferreira et al. 2015). In the CNS, it can be expressed by activated microglia, astrocytes, neurons, and choroid plexus in response to different inflammatory stimuli (Marques et al. 2008; Ip et al. 2011; Rathore et al. 2011; Xing et al. 2014; Jha et al. 2015).

To extend the knowledge of the different astroglial subpopulations, we performed an immunohistochemical study to investigate the expression levels and specificity of different astrocytic markers at different stages of de- and remyelination in distinct brain regions, using the well-established cuprizone animal model for toxic demyelination (Matsushima and Morell 2001; Skripuletz et al. 2011; Goldberg et al. 2015; Gudi et al. 2014). Cuprizone [bis(cyclohexanone)oxaldihidrazone] is a cupper chelating agent, which, when supplemented to normal rodent chow (0.2% w/w), leads to oligodendroglial cell death, micro- and astrogliosis, and subsequent demyelination, resembling the histopathological changes found in the third pattern of MS lesions according to the conventional classification I–IV based on complement activation, presence of IgG and periferal immune cells, oligodendrocyte dystrophy, and loss of myelin (Lucchinetti et al. 2000; Lassmann et al. 2007). Since remyelination directly follows the termination of the cuprizone diet, this animal model is particularly useful in the study of astrocyte involvement during regeneration processes (Blakemore 1973; Stidworthy et al. 2003; Steelman et al. 2012; Skripuletz et al. 2013).

## Materials and methods

### Animals

Male *C57BL/6* mice, 8–10 weeks old, were purchased from Charles River Laboratories (Sulzfeld, Germany). Animals underwent routine cage maintenance once a week and were microbiologically monitored according to Federation of European Laboratory Animal Science Associations recommendations (Nicklas et al. 2002). All research procedures were approved by the Review Board for the Care of Animal Subjects of the district government and performed according to international guidelines on the use of laboratory animals.

### Induction of demyelination and tissue processing

Experimental toxic demyelination was induced by feeding 8–10-week-old male *C57BL/6* mice with 0.2% (w/w) cuprizone (bis(cyclohexanone)oxaldihydrazone, Sigma-Aldrich) added to a ground standard rodent chow (Skripuletz et al. 2011). To investigate acute demyelination, the cuprizone diet was maintained for 5 weeks. For remyelination, animals were returned to normal chow for an additional 2 weeks. Brains were collected at different time points (3 weeks—early demyelination, 5 weeks—maximal demyelination, 5.5 weeks—early remyelination, 7 weeks—late remyelination). Tissue processing was performed as described previously (Gudi et al. 2011; Benardais et al. 2014). The anesthetic procedure was performed in accordance with the appropriate animal study approval (AZ: 12/0866). Mice were injected intraperitoneally with a lethal overdose of a mixture of ketamine (CP-Pharma) and 2% Rompun^®^ (xylazine hydrochloride, Bayer) and perfused postmortem transcardially with 4% paraformaldehyde (PFA, Merck).

For immunohistochemistry, brains were post-fixed in 4% PFA and embedded in paraffin. Seven-micrometer serial coronal sections were cut on a bright rotary microtome (RM2245, Leica) from −0.82 mm bregma to −1.70 mm bregma. A group size of four to six animals was evaluated at each time point.

### Immunohistochemistry

For immunohistochemistry, paraffin-embedded brain sections were dewaxed and heat-unmasked in 10 mM citrate buffer (pH 6.0). For immunofluorescence staining, sections were blocked with phosphate-buffered saline (PBS) containing 10% normal goat serum and 0.1% Triton X-100. Slides were then incubated overnight at 4 °C with the following primary antibodies: myelin proteolipid protein (PLP; mouse IgG2a, 1:500, Serotec, Bio-Rad), glial fibrillary acidic protein (GFAP; mouse IgG1, 1:200, Merck Millipore), (GFAP; rabbit, 1:200, Dako), vimentin (VIM, rabbit IgG,1:500, Abcam), aldehyde dehydrogenase (ALDH1L1, mouse IgG, 1:1000, Merck Millipore), calcium-binding protein B (S100B; rabbit IgG, 1:500, Abcam), adenomatous polyposis coli (APC, clone CC1; mouse IgG2b, 1:200, Calbiochem, Merck Millipore), lipocalin 2 (LCN2; goat IgG, 1:200, R&D), ricinus communis agglutinin (RCA-1, 1:1000, Vector Laboratories), OLIG2 (rabbit IgG,1:500, Chemicon, Merck Millipore) (Table 1). Sections were then washed with PBS and incubated for 1 h with the appropriates secondary antibodies: anti-mouse immunoglobulin G1 Alexa-488 conjugated (1:500, Thermo Fisher Scientific), anti-mouse immunoglobulin G2b Alexa-555 conjugated (1:500, Thermo Fisher Scientific), anti-rabbit immunoglobulin G (H+L) Alexa-488 conjugated (1:500, Thermo Fisher Scientific), anti-rabbit immunoglobulin G (H+L) Alexa-555 conjugated (1:500, Thermo Fisher Scientific), anti-mouse immunoglobulin G (H+L) Alexa-488 conjugated (1:500, Thermo Fisher Scientific), anti-mouse immunoglobulin G (H+L) Alexa-555 conjugated (1:500, Thermo Fisher Scientific), and anti-goat immunoglobulin G (H+L) Alexa-555 conjugated (1:500, Thermo Fisher Scientific) (Table 2). Slides were mounted with Mowiol (Calbiochem) and counterstained with DAPI (Invitrogen) for immunofluorescence staining (Salinas Tejedor et al. 2015).

**Table 1:** List of primary antibodies used in this study

Antigen	Host	Dilution	Purchase number, Clone	Supplier	RRID	Target cells
ALDH1L1	Mouse, IgG1k	1:1000	MABN495, Clone N103/39	Merck Millipore	AB_2687399	Astrocytes
APC-CC1	Mouse, IgG2b	1:200	OP80 Clone CC1	Merck Millipore, Calbiochem,	AB_2057371	Oligodendrocytes, astrocytes
GFAP	Rabbit, polyclonal	1:200	Z0334	Agilent Dako	AB_10013382	Astrocytes
GFAP	Mouse, IgG1	1:200	MAB 360, Clone GA5	Merck Millipore	AB_11212597	Astrocytes
LCN2	Goat, polyclonal	1:200	AF1857	R&D	AB_355022	Astrocytes, microglia, neutrophils
NeuN	Rabbit, polyclonal	1:500	PA5-78499	Invitrogen, Thermo Fisher Scientific	AB_2736206	Neurons (cell bodies)
PLP	Mouse IgG2a	1:500	MCA839G	Bio-Rad	AB_2237198	Myelin
RCA-1	Lectin	1:1000	FL1081	Vector Laboratories	AB_2336708	Activated microglia
S100B	Rabbit, monoclonal, animal-free	1:500	ab52642	Abcam	AB_882426	Astrocytes, oligodendrocytes
OLIG2	Rabbit, polyclonal	1:500	AB9610	Merck Millipore	AB_570666	Oligodendrocytes
Vimentin	Rabbit, monoclonal	1:500	ab92547	Abcam	AB_10562134	Activated astrocytes

**Table 2:** List of secondary antibodies used in this study

Antigen	Host	Dilution	Purchase number	RRID	Supplier
Anti-mouse IgG (H+L) Alexa Fluor 488	Goat, polyclonal	1:500	A-11001	AB_2534069	Thermo Fisher Scientific
Anti-mouse IgG1 Alexa Fluor 488	Goat, polyclonal	1:500	A-21121	AB_2535764	Thermo Fisher Scientific
Anti-mouse IgG2b Alexa Fluor 555	Goat, polyclonal	1:500	A-21147	AB_2535783	Thermo Fisher Scientific
Anti-mouse IgG (H+L) Alexa Fluor 555	Goat, polyclonal	1:500	A-21422	AB_2535844	Thermo Fisher Scientific
Anti-goat IgG (H+L) Alexa Fluor 555	Donkey, polyclonal	1:500	A-21432	AB_2535853	Thermo Fisher Scientific
Anti-rabbit IgG (H+L) Alexa Fluor 555	Goat, polyclonal	1:500	A-21428	AB_2535849	Thermo Fisher Scientific
Anti-rabbit IgG (H+L) Alexa Fluor 488	Goat, polyclonal	1:500	A-11008	AB_143165	Thermo Fisher Scientific

### Quantification of astroglial reactions and expression of different astroglial markers

Paraffin brain sections were stained with different astroglial markers. All images were acquired with an Olympus BX61 microscope **(**Olympus, Japan) and an Olympus DP72 camera at magnification of ×200 and ×400 in 1024 × 1024 resolution (Skripuletz et al. 2011; Salinas Tejedor et al. 2015). The cellSens Dimension software **(**Olympus, Japan) was used to qualify the staining and count the cells in the regions of interest. Immunopositive cells were analyzed within the corpus callosum (central and lateral), cerebral cortex, and hippocampal areas CA3 and dentate gyrus. Counted cells were expressed as number of cells/mm^2^.

### RNA isolation and quantitative RT-PCR

The complete corpus callosum, cerebral cortex, and hippocampus were dissected manually from whole brains under a light microscope. Total RNA was extracted according to the manufacturer’s recommendations using the RNeasy Mini Kit (Qiagen). RNA concentrations were measured with the NanoDrop 2000c spectrophotometer (Thermo Fisher Scientific), and complementary DNA (cDNA) was synthesized using the High Capacity cDNA Reverse Transcription Kit (Applied Biosystems, Thermo Fisher Scientific). Real-time quantitative analysis was performed with the StepOne Real-Time PCR System and TaqMan assays (Applied Biosystems, Thermo Fisher Scientific). The gene expression of *Gfap*, *Vim*, *Lcn2*, *Aldh1l1*, and *S100b* (Table 3) was investigated in the corpus callosum, cortex, and hippocampus at three time points (weeks 3, 5, and 5+2). Differences in gene expression between cuprizone-exposed animals and untreated controls were analyzed with the ΔΔCt method (*n* = 4–7). The probes were normalized to hypoxanthine phosphoribosyltransferase 1 (*Hprt1*), which had been identified as a stable reference gene in a cuprizone animal model (Gudi et al. 2011; Skripuletz et al. 2013).

**Table 3:** List of TaqMan assays used in this study

Gene name	Assay ID
*Gfap*	4331182 Mm01253033_m1
*Vim*	4331182 Mm01333430_m1
*Lcn2*	4331182 Mm01324470_m1
*Aldh1l1*	4331182 Mm03048957_m1
*S100b*	4331182 Mm00485897_m1
*Hprt1*	4331182 Mm00446968_m1

### Statistical analysis

Statistical analysis was performed using the Kruskal–Wallis test, followed by the Dunn test. All data are given as arithmetic means ± standard error of the mean (SEM). *P* values of the different Kruskal–Wallis tests are given in the results, while group comparisons derived from post hoc analysis are provided in the figures. In all cases, significant effects versus controls are indicated by rhombs (#*p* < 0.05, ##*p* < 0.01, and ###*p* < 0.001) and significant effects versus different time points are indicated by a line and asterisks (*p < 0.05, **p < 0.01, and ***p < 0.001).

## Results

### Cuprizone causes profound and region-specific demyelination in C57BL/6 mice

**Fig. 1:**
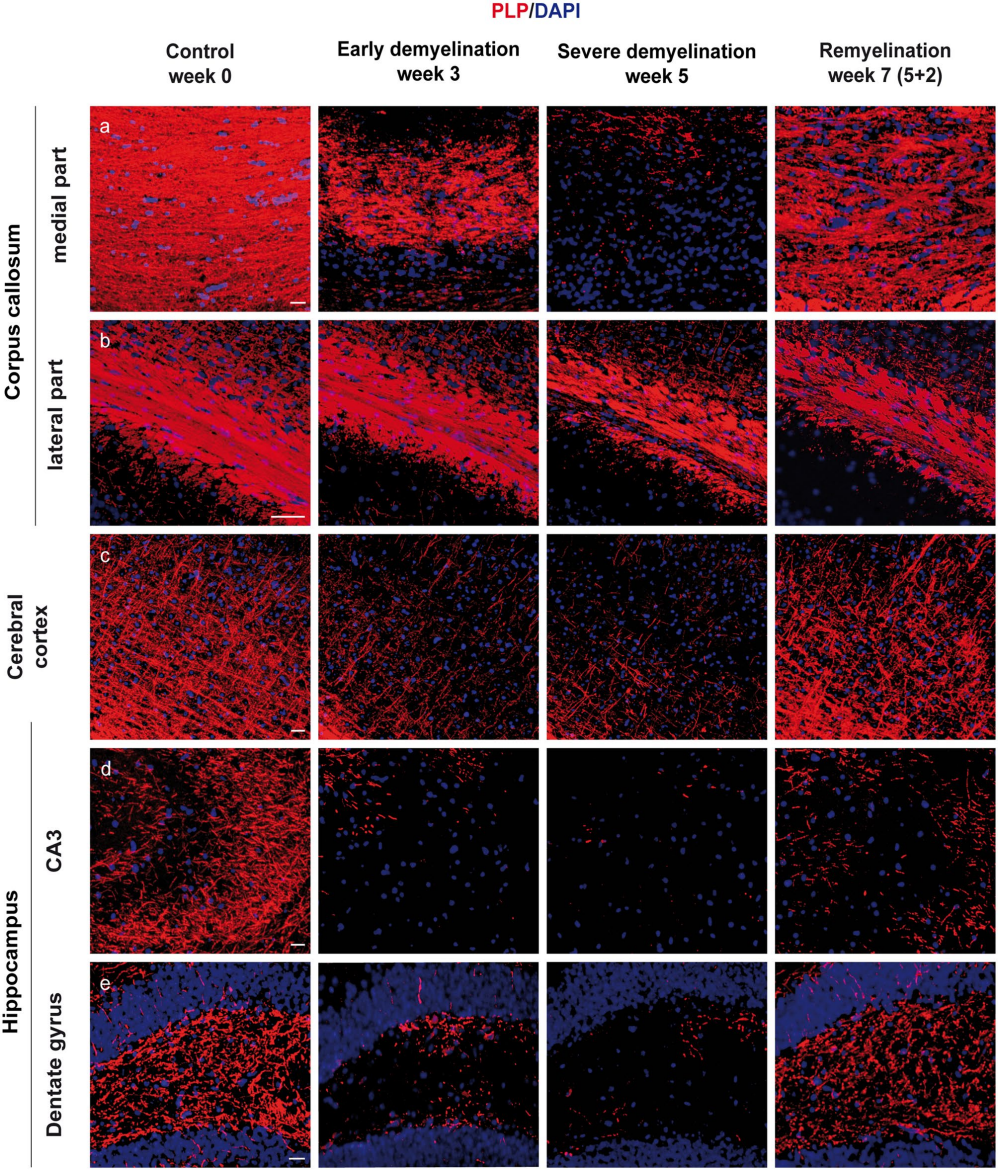
The course of de- and remyelination in representative brain regions of C57BL/6 mice exposed to cuprizone. Double immunofluorescence staining with antibodies against the myelin marker proteolipid protein (PLP) was performed to visualize the course of de- and remyelination in the midline (**a**) and lateral parts of corpus callosum (**b**), cerebral cortex (**c**) (represented layers V–VI), and hippocampal areas CA3 (**d**) and dentate gyrus (**e**) and in C57BL/6 mice exposed to cuprizone (nuclei were counterstained with DAPI). Representative pictures in the first column show the corresponding regions with intact myelin of untreated animals (week 0). Cuprizone feeding provoked a progressive loss of myelin (middle columns), with nearly complete demyelination at week 5 in all studied brain regions except for the lateral part of the corpus callosum, where only partial demyelination occurred (third column). Finally, re-expression of PLP could be observed 2 weeks after cuprizone withdrawal during remyelination (last column). Scale bars: 20 µm (**a**, **c**, **d**, **e**); 50 µm (**b**)

De- and remyelination in C57BL/6 mice was induced by addition of 0.2% cuprizone to normal mice chow for 5 weeks, following the same protocols previously used in our lab (Skripuletz et al. 2011; Gudi et al. 2014). Immunofluorescence assays with antibodies against the proteolipid protein (PLP) showed a vast loss of myelin in the midline of the corpus callosum, cerebral cortex, hippocampal region CA3, and the dentate gyrus beginning at week 3 of treatment, as well as profound to almost complete demyelination of the same regions at week 5 (Fig. 1). Moreover, we also observed a certain resistance to cuprizone-induced demyelination in the lateral corpus callosum, which is in line with other works (Schmidt et al. 2013; Tagge et al. 2016). Finally, termination of the cuprizone diet at week 5 resulted in a recovery of myelin during the following 2 weeks (Fig. 1).

### Astrocyte-specific genes are differentially regulated in different brain regions during cuprizone-induced demyelination

**Fig. 2:**
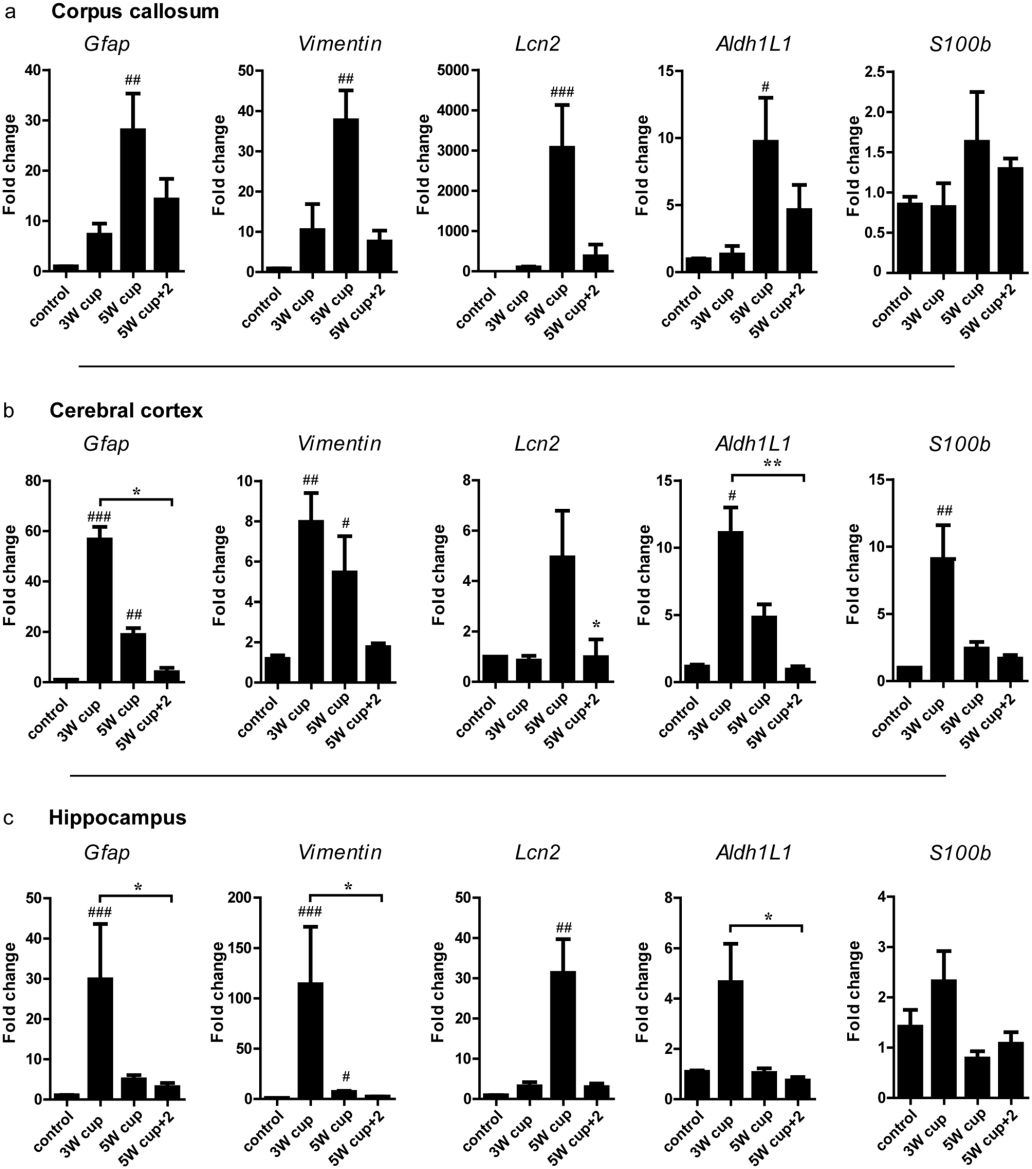
mRNA expressions of *Gfap*, *Vim, Aldh1L1, S100b*, and *Lcn2* in the corpus callosum, cerebral cortex, and hippocampus during cuprizone-induced de- and remyelination. Graphs show the mRNA expression of different astrocytic markers during the course of de- and remyelination in the corpus callosum (**a**) (*Gfap*: *p* = 0.0018, *vimentin*: *p* = 0.0056, *Aldh1l1*: *p* = 0.0163, *Lcn2*: *p* = 0.0014, *S100b*: not significant), cerebral cortex (**b**) (*Gfap*: *p* = 0.0008;* vimentin*: *p* = 0.024;* Aldh1l1*: *p* = 0.0018;* Lcn2*: *p* = 0.0183; *S100b*: *p* = 0.0034), and hippocampus (**c**) (*Gfap*: *p* = 0.0009,* vimentin*: *p* = 0.0003, *Aldh1l1*: *p* = 0.0294,* Lcn2*: *p* = 0.0033, *S100beta*: not significant). 3W cup: beginning of demyelination, week 3; 5W cup: severe demyelination, week 5; 5W cup+2: remyelination, week 7. Bar charts show mean values and SEMs. Significant effects versus controls are indicated by rhombs (#*p* < 0.05, ##*p* < 0.01, and ###*p* < 0.001) and significant effects versus different time points are indicated by a line and asterisks (*p < 0.05, **p < 0.01, and ***p < 0.001), *n* = 4–7.

First, we analyzed mRNA expression of astrocyte-specific genes in different brain regions by applying the real-time PCR technique. In the cerebral cortex, corpus callosum, and hippocampus, most astrocytic genes studied were significantly regulated during cuprizone-induced demyelination as compared to untreated controls, with the exception of *S100b* in the corpus callosum and hippocampus, *Lcn2* in the cortex, and *Aldh1l1* in the hippocampus (Fig. 2).

In general, *Gfap*, *Vim*, and *Aldh1l1* showed similar gene expression patterns; however, there were clear regional differences (Fig. 2). Whereas in the corpus callosum the expression of *Gfap*, *Vim*, and *Aldh1l1* peaked at week 5 during severe demyelination (fold changes: *Gfap* ca. 30, *Vim* ca. 40, and *Aldh1l1* ca. 10), in the cerebral cortex and hippocampus the highest expression of these genes was already observed at the beginning of demyelination, at week 3 (fold changes for cerebral cortex: *Gfap* ca. 60, *Vim* ca. 8, and *Aldh1l1* ca. 12; fold changes for hippocampus: *Gfap* ca. 32, *Vim* ca. 120, and *Aldh1l1* ca. 5) (Fig. 2). Compared with control levels, the greatest rise in *Gfap* mRNA expression was observed in the cerebral cortex. *Vim* mRNA expression was highest in the hippocampus, whereas *Aldh1l1* mRNA expression increased equally in all regions examined. During remyelination, the expression of *GFAP*, *Vim*, and *Aldh1l1* mRNA decreased (Fig. 2). *S100b* was only slightly regulated after the challenge with cuprizone (max fold changes for *S100b* in corpus callosum ca. 1.8, cerebral cortex ca. 10, and hippocampus ca. 2.5) (Fig. 2). Again, we could see the same tendency that we observed for *Gfap*, *Vim*, and *Aldh1l1* gene expression. In the gray matter structures, such as cortex and hippocampus, *S100b* was upregulated already at the beginning of the demyelination (Fig. 2b and c). In the corpus callosum, the expression of *S100b* was only minimally increased up to week 5 but it failed to be significant (Fig. 2a). Only *Lcn2* showed the same regulation patterns in all three brain areas studied (Fig. 2), reaching its mRNA expression peak at severe demyelination. However, there were huge regional differences in mRNA expression levels. The upregulation of *Lcn2* in the corpus callosum at week 5 in comparison to the basic expression was 10–100 times higher (fold change: ca. 3500) than in the cerebral cortex (fold change: ca. 5) and hippocampus (fold change: ca. 35), respectively.

### Expression of vimentin and GFAP occurs simultaneously during de- and remyelination in the cuprizone model

**Fig. 3:**
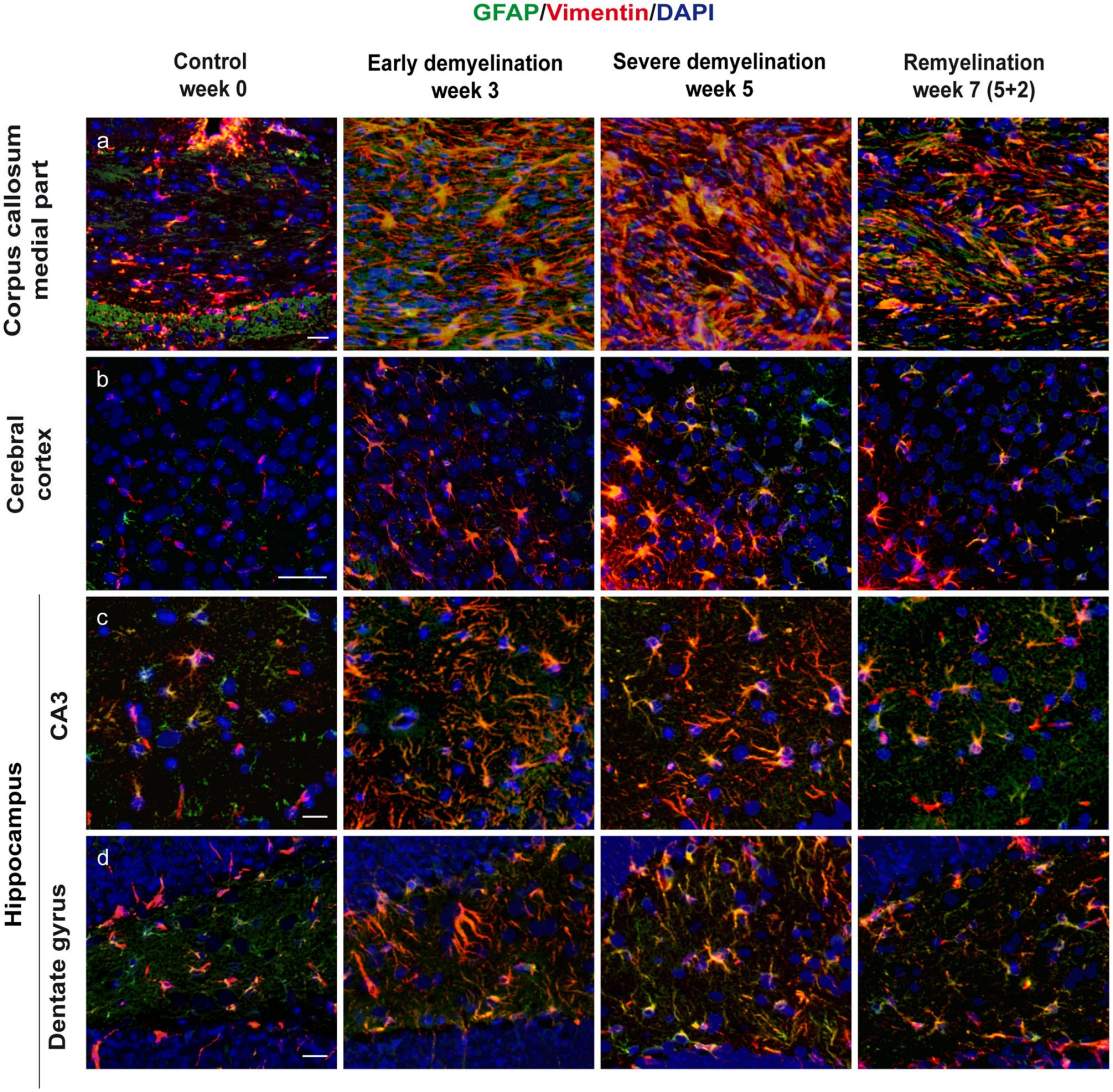
Immunohistochemical analysis of gliosis with astrocytic markers VIM and GFAP. Representative pictures demonstrate immunofluorescence double staining with vimentin (red) and GFAP (green) of the central corpus callosum (**a**), cerebral cortex (represented layers IV–VI) (**b**), and hippocampal areas CA3 (**c**) and dentate gyrus (**d**) during the course of demyelination and remyelination in C57BL/6 mice exposed to cuprizone (nuclei were counterstained with DAPI). Representative images in the left column represent the equivalent brain regions of untreated mice (week 0). During demyelination, astrocytes become reactive and re-express GFAP and vimentin. An extensive co-localization of both markers was found in all examined areas. Overexpression of both markers continued during the process of remyelination, as we can observe in the right column at week 7 (2 weeks after cuprizone withdrawal). Scale bars: 20 µm (**a**, **c**, **d**); 50 µm (**b**)

The upregulation of intermediate filaments is a well-known astrocytic response to pathological conditions (Eng and Ghirnikar 1994; Pekny and Pekna 2014). As judged by immunohistochemistry, GFAP co-localized with VIM (Fig. 3). Their expression was simultaneously upregulated and followed similar temporal patterns in the white and gray matter of cuprizone-fed mice (Fig. 3). In addition, a scattered population of small round bipolar cells with exclusive expression of vimentin was visible throughout the gray matter along the endothelium of blood vessels at all time points. The cessation of cuprizone caused downregulation of astrocytic intermediate filament proteins with slight amelioration of fluorescence intensity (Fig. 3).

### ALDH1L1 is expressed on astrocytes in CNS white and gray matter and co-localizes with OLIG2 in a small cell population

**Fig. 4:**
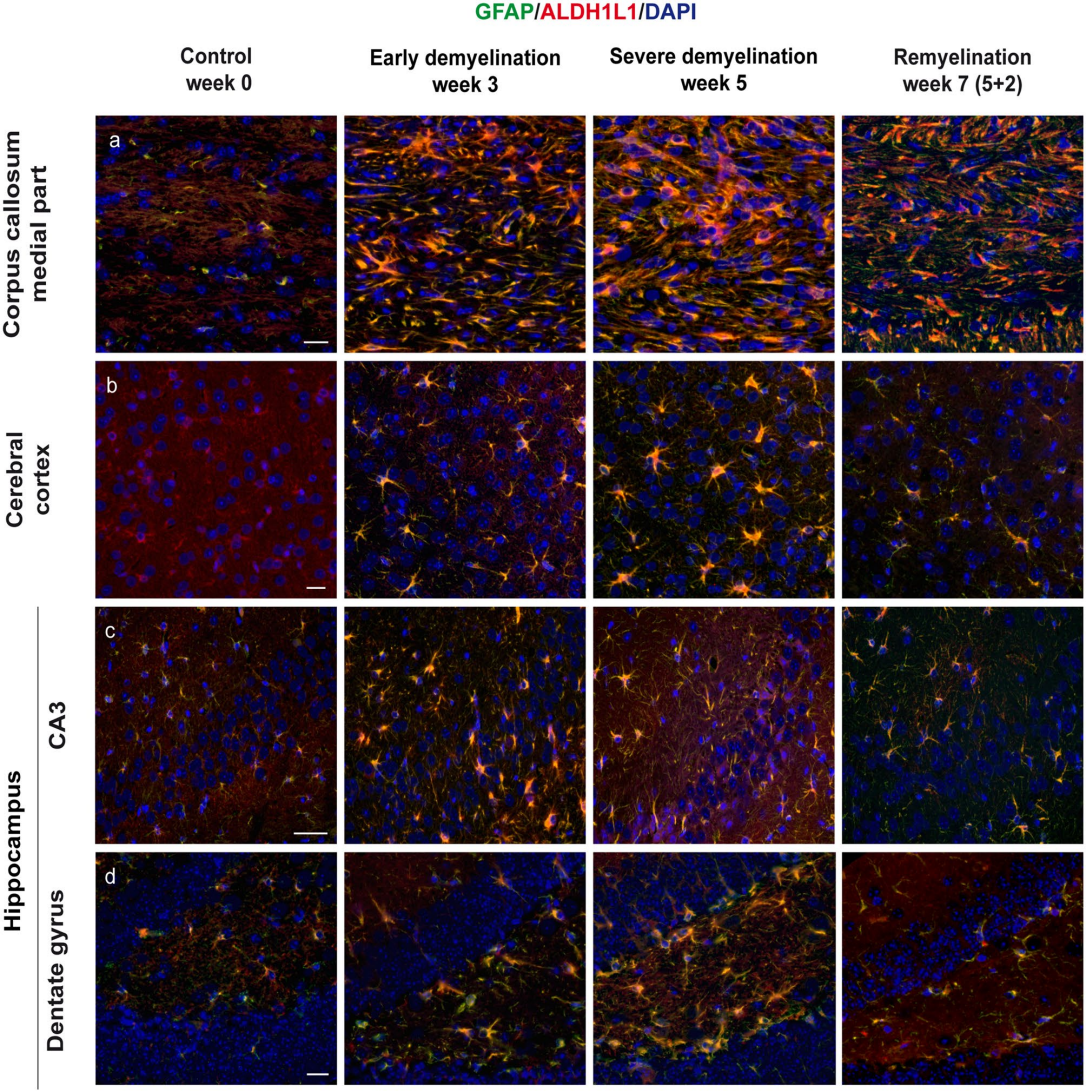
Immunohistochemical analysis of gliosis with astrocytic markers ALDH1L1 and GFAP. Immunofluorescence double staining depicting GFAP (green) and ALDH1L1 (red) in the central corpus callosum (**a**), cerebral cortex (represented layers V–VI) (**b**), and hippocampal areas CA3 (**c**) and dentate gyrus (**d**) during the course of demyelination and remyelination in C57BL/6 mice exposed to cuprizone (nuclei were counterstained with DAPI). The left column represents the equivalent brain regions of untreated mice. ALDH1L1 was detected in protoplasmic astrocytes of the cerebral cortex under physiological conditions, albeit at very low levels (**b**). Under cuprizone feeding, both markers became upregulated (middle columns). Overexpression of both markers remained during the process of remyelination, as we can observe in the right column at week 7 (2 weeks after cuprizone withdrawal). Scale bars: 20 µm (**a**, **c**, **d**); 50 µm (**b**)

**Fig. 5:**
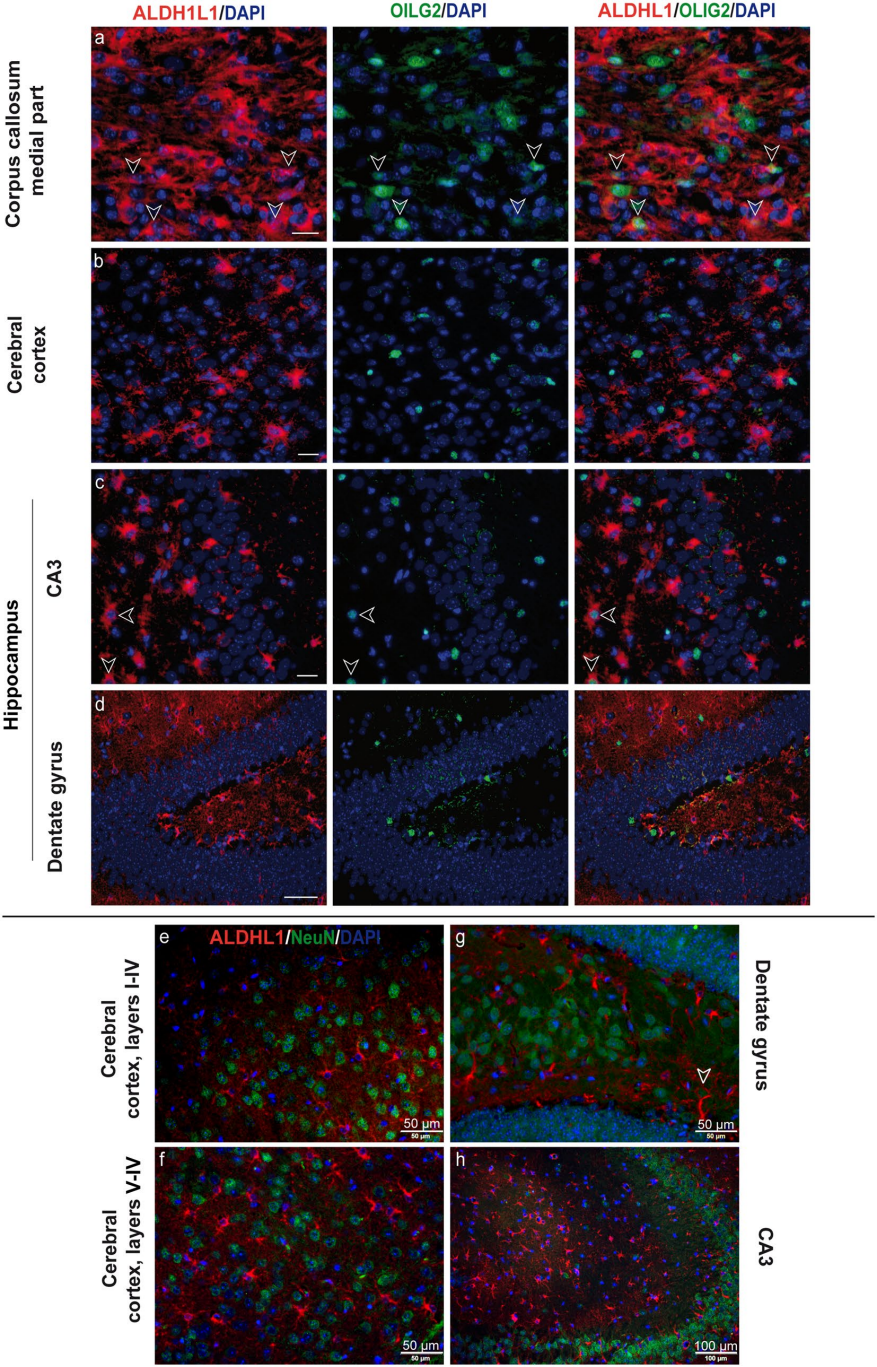
Immunohistochemical analysis of gliosis with astrocytic markers ALDH1L1 and OLIG2. Representative images show ALDH1L1 (red)/OLIG2 (green) double stained section of the central corpus callosum (**a**), cerebral cortex (represented layers V–VI) (**b**), and hippocampal areas CA3 (**c**) and dentate gyrus (**d**) after 5-week cuprizone exposure (nuclei were counterstained with DAPI). Co-localization of both markers was detected in the small population of cells in the corpus callosum and hippocampal area CA3 (arrowheads). Double staining with NeuN and ALDH1L1 antibodies in gray matter areas are shown in panels (**e**–**h**). In general, ALDH1L1 did not show concordance with NeuN in the brain regions examined, with the exception of the gyrus dentate, where a co-localization of these two markers (arrowhead) was sporadically detected. NeuN was not detected in the corpus callosum (data not shown). Scale bars: 20 µm (**a**, **b**, **c**); 50 µm (**d**, **e**, **f**, **g**) 100 µm (**h**)

The L1 member of the aldehyde dehydrogenase 1 family (ALDH1L1) has recently been introduced as a broad and specific astrocytic marker (Cahoy et al. 2008; Yang et al. 2011). To analyze its expression dynamics during cuprizone-induced de- and remyelination, we conducted double immunofluorescence studies with ALDH1L1 and GFAP. Indeed, cuprizone feeding triggered simultaneous upregulation of both markers, with extensive co-localization in all brain regions studied. Furthermore, ALDH1L1 proved to be a suitable marker for labeling protoplasmic astrocytes in cerebral cortex gray matter and the hippocampus under physiological conditions, in spite of its low expression levels (Fig. 4). Two weeks after cuprizone withdrawal, we observed a marked decrease in ALDH1L1 expression in all brain areas examined. Corresponding to previous works, complementary assays with the oligodendrocyte marker OLIG2 evidenced a transient co-expression with ALDH1L1 in a small, star-shaped cell population in the corpus callosum and the external hippocampal layer CA3 only at week 5 of cuprizone administration (Fig. 5, arrowheads) (Yang et al. 2011; Salinas Tejedor et al. 2015) The co-localization of OLIG2 and ALDH1L1 demonstrated in this study may suggest that these ALDH1L/1+/OLIG2+ cells represent a subpopulation of progenitor cells in the postnatal brain that could give rise to both astrocytes and oligodendrocytes.

Given the knowledge that ALDH1L1 can be expressed by postnatal neural stem cells in vivo, we performed ALDH1L1/NeuN staining to assess the expression of ALDH1L1 in neuronal lineage cells (Foo and Dougherty 2013). In general, ALDH1L1 did not show concordance with NeuN in the brain regions examined, with the exception of the gyrus dentate, where we indeed occasionally detected co-localization of these two markers (Fig. 5).

### S100B is intensively co-expressed with GFAP in the cerebral cortex and hippocampal CA1–CA3 astrocytes, but is lacking in a population of GFAP+ astrocytes in the corpus callosum and the hippocampal dentate gyrus

**Fig. 6:**
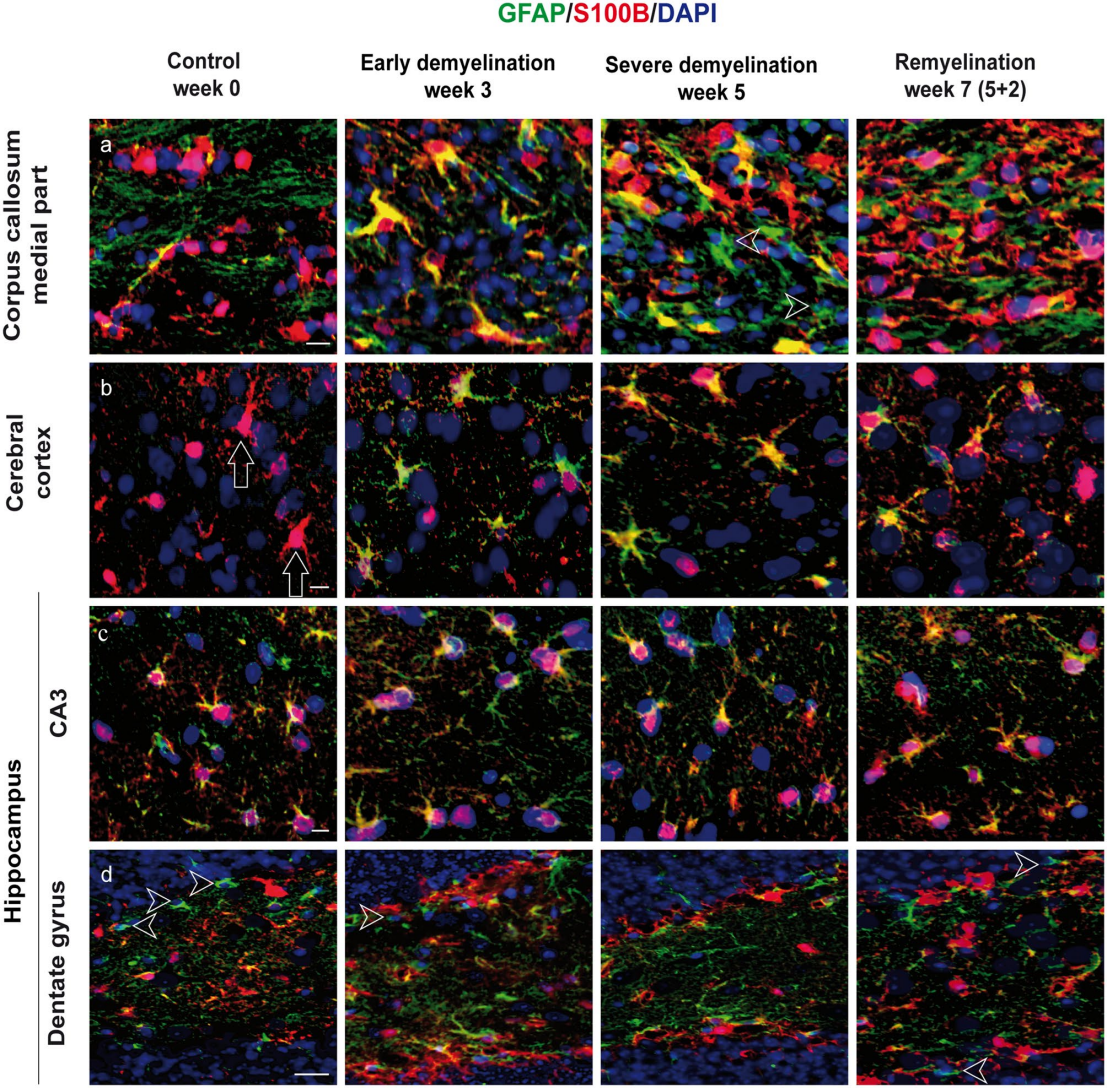
Immunohistochemical analysis of gliosis with astrocytic markers GFAP and S100B. Representative pictures demonstrate immunofluorescence co-staining with GFAP (green) and S100B (red) of the central corpus callosum (**a**), cerebral cortex (represented layers V–VI) (**b**), and hippocampal areas CA3 (**c**) and dentate gyrus (**d**) during the course of demyelination and remyelination in C57BL/6 mice exposed to cuprizone (nuclei were counterstained with DAPI). The left column shows the equivalent brain regions of untreated mice with multiple single S100B+ cells that represent oligodendrocytes. In addition, star-shaped cells corresponding to protoplasmic astrocytes were stained with S100B in the cerebral cortex of control animals (B, small arrows). Cuprizone feeding provoked a marked reduction in the number of single S100B+ cells parallel to an increase of double S100B+/GFAP+ cells (middle columns). Furthermore, a small population of S100B−/GFAP+ cells was detected in the hippocampal region dentate gyrus and corpus callosum (arrowheads). Finally, during remyelination, a progressive increase in the number of single S100B+ cells occurred (right column). Scale bars: 10 µm (**a**, **b**, **c**); 20 µm (**d**)

**Fig. 7:**
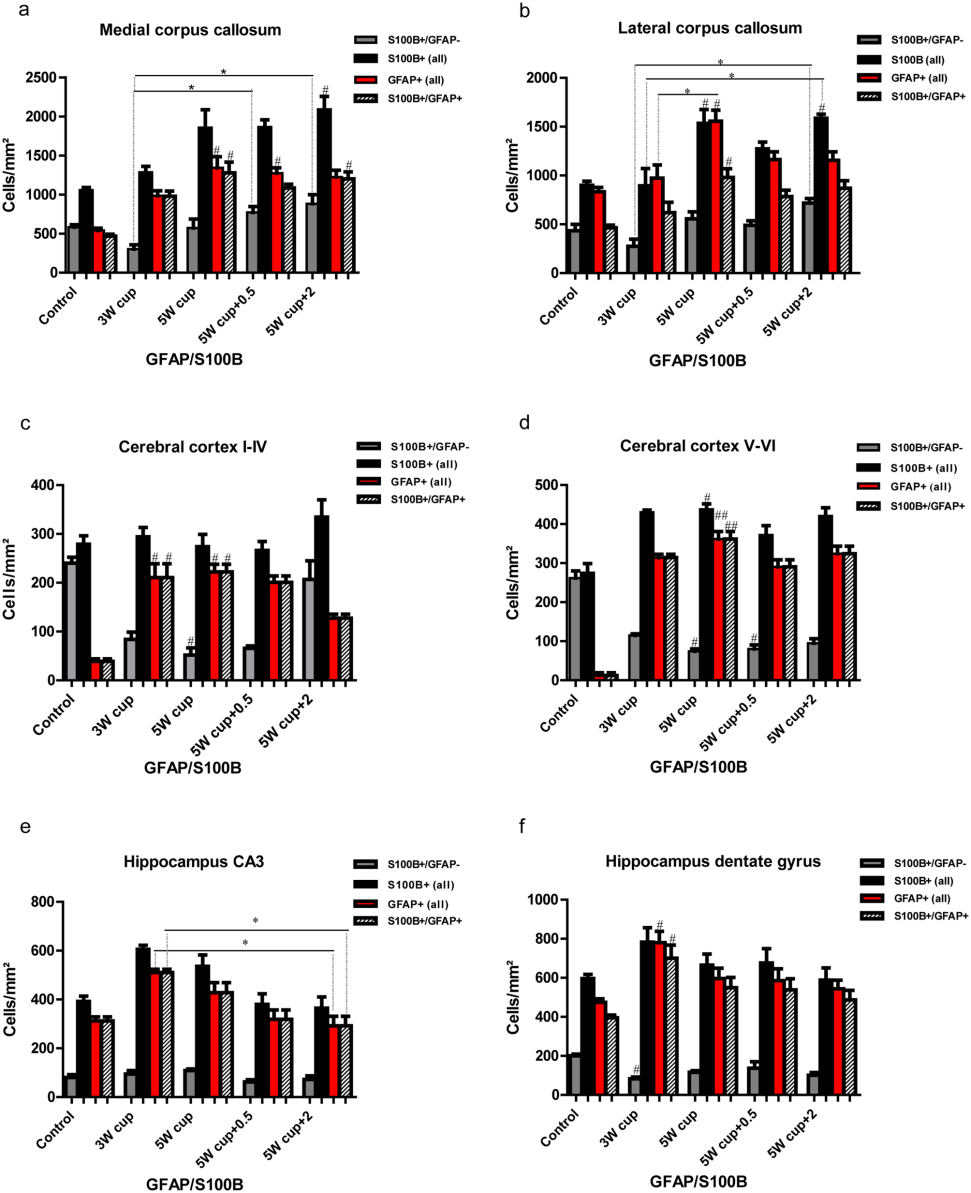
Expression dynamics of S100B and GFAP during the course of de-and remyelination. Graphs represent the qualitative analysis of GFAP+ (red bars), S100B+ (black bars), and GFAP+/S100B+ cells (striped bars) during the course of de- and remyelination in the different brain regions (3W cup: beginning of demyelination, week 3; 5W cup: severe demyelination, week 5; 5W cup+0.5: early remyelination, week 5.5; 5W cup+2: remyelination, week 7). Graphics **a** and **b** describe the effect of cuprizone on astrocytes (all GFAP+ and S100B+/GFAP+ cells) in the medial and lateral part of corpus callosum, **c** and **d** in different cortical layers, **e** and **f** in the hippocampus. During cuprizone treatment astrocytes proliferate, reaching a peak at week 5 (red and striped columns). Differences between black and striped columns show the ratio/relation between all S100B+ cells and GFAP+ astrocytes. The difference between the red and striped columns corresponds to a short population of astrocytes lacking on S100B immunoreactivity. There are some regional differences in the expression of S100B by GFAP+ astrocytes in both control and cuprizone-treated animals. Bar charts show mean values and SEMs. Significant effects versus controls are indicated by rhombs (*#p* < 0.05,* ##p* < 0.01, and *###p* < 0.001) and significant effects versus different time points are indicated by a line and asterisks (*p < 0.05, **p < 0.01, and ***p < 0.001), *n* = 4.

S100B is a calcium-, zinc-, and copper-binding protein mostly expressed by astrocytes, with autocrine and paracrine effects on neurons, glia, and microglial cells (Rothermundt et al. 2003; Steiner et al. 2007). This antigen has also been detected in oligodendrocytes and cortical neurons, depending on isoform and antibody (Rickmann and Wolff 1995). Here, we investigated the cellular expression of S100B throughout the white and gray matter immunohistochemically in C57BL/6 mice exposed to cuprizone. To address the astrocytic specificity of S100B during cuprizone-induced demyelination and subsequent remyelination, we performed double immunofluorescence staining with S100B and GFAP. In the corpus callosum midline of untreated animals, only 45% of the S100B+ cells co-localized with GFAP, although almost all GFAP+ astrocytes were positive for S100B. In contrast, a huge population of GFAP+/S100B− cells (up to 44% of all GFAP+ cells) was found in the lateral corpus callosum. In the hippocampal region CA3 of control animals, 79% of all S100B cells co-localized with GFAP; in the dentate gyrus it was only 66%. On the other hand, almost all GFAP+ astrocytes in these regions expressed S100B. Immunohistochemically, the GFAP expression in cerebral protoplasmic astrocytes of untreated control animals was not detectable; thus co-localization of both markers in the cerebral cortex was barely visible (Figs. 6b and 7c and d). Conversely, S100B was broadly detected in the cerebral cortex in physiological circumstances.

Cuprizone induces simultaneous upregulation of both GFAP and S100B in all brain regions studied (Fig. 7). At weeks 3 and 5 of cuprizone-induced demyelination, 76% and 69%, respectively, of all S100B+ cells co-localized with GFAP in the corpus callosum midline (Figs. 6 and 7a); simultaneously, almost all GFAP+ astrocytes (99% and 95%) expressed S100B (Figs. 6 and 7a). In contrast, in the lateral corpus callosum, high amounts of GFAP+ astrocytes (up to 37%) were negative for S100B, whereas 69% and 64% of all S100B+ cells at weeks 3 and 5 co-expressed GFAP, respectively. In the cortex, S100B and GFAP immunostaining overlapped up to 73% at week 3, and 83% at week 5 of cuprizone treatment (Figs. 6, 7c and d), whereas all GFAP-positive astrocytes co-express S100B. Similar values were found in both hippocampal areas studied (Fig. 7e and f). A fluctuating population (8–17%) of GFAP+/S100B− astrocytes with small somata and a bipolar morphology was identified in the subgranular layer of the dentate gyrus at all time points (Figs. 6 and 7f).

During remyelination (week 7), only 58% of S100B+ cells co-localized with GFAP in the corpus callosum midline (Fig. 7a), and 55% of all S100B+ cells were positive for GFAP in the lateral corpus callosum. Again, numerous GFAP+ astrocytes did not express S100B here. In the cortex, overlap of S100B and GFAP decreased to 38% at week 7 (layers I–IV) (Figs. 6, 7c and d). In contrast, 80 and 83% overlay were observed in both the hippocampal CA3 and dentate gyrus (Fig. 7e and f).

### S100B co-localizes with oligodendroglial marker APC-CC1 during de- and remyelination, pointing to the distinct population of oligodendrocytes

**Fig. 8:**
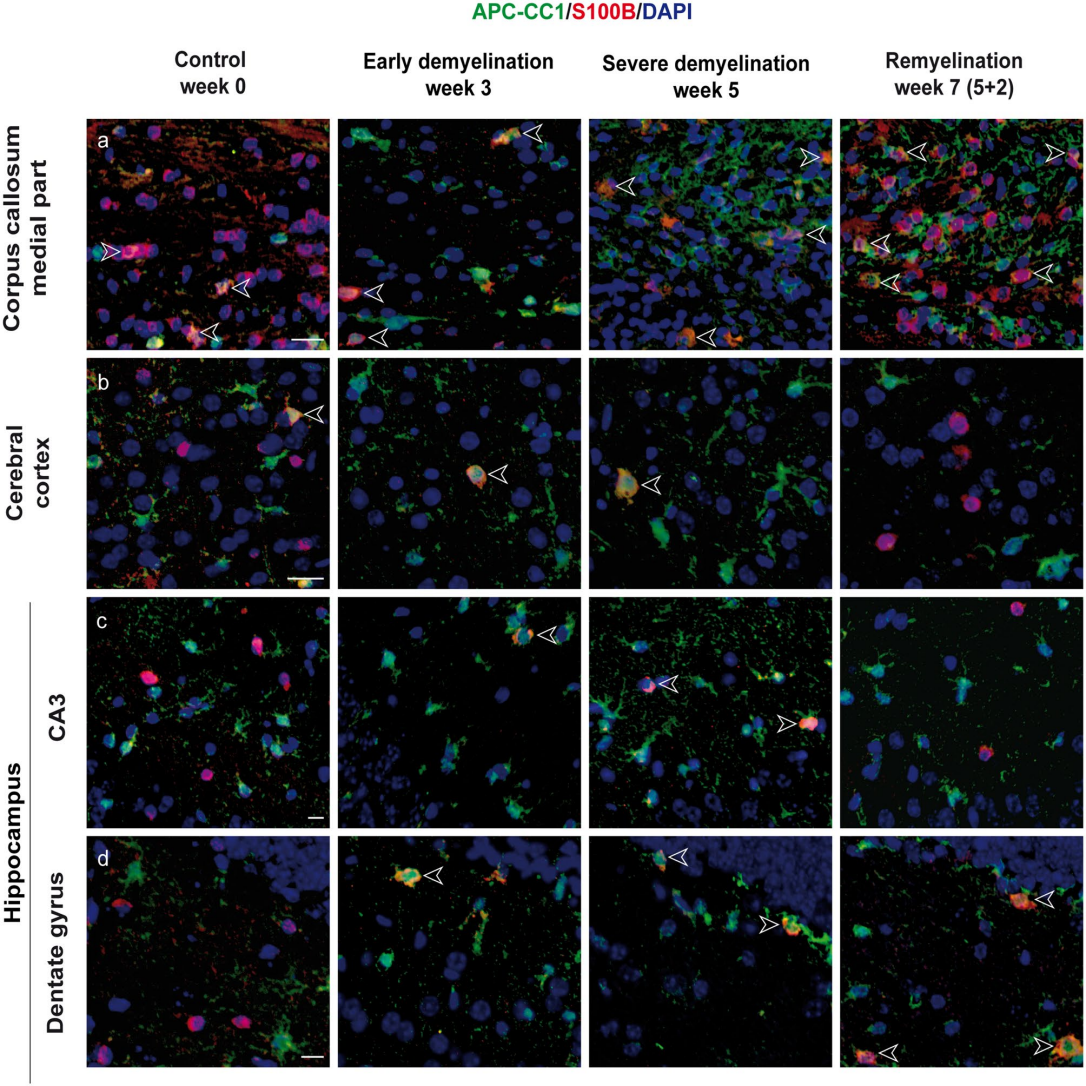
Immunohistochemical analysis of expression of S100B and APC-CC1 in oligodendrocytes. Representative images show immunofluorescence staining of S100B (green) and APC-CC1 (red) in the central corpus callosum (**a**), cerebral cortex (represented layers V–VI) (**b**), and hippocampal areas CA3 (**c**) and dentate gyrus (**d**) during the course of demyelination and remyelination in C57BL/6 mice exposed to cuprizone (nuclei were counterstained with DAPI). The left column corresponds to the equivalent brain regions of untreated mice containing a high number of single APC-CC1+ and double S100B+/APC-CC1+ cells, which represent oligodendrocytes. Cuprizone diet provoked a severe demyelination in all studied brain regions reflected by a significant diminution of the APC-CC1+ oligodendrocytes numbers. Two weeks after cuprizone withdrawal, a significant recovery of myelin was mirrored by a progressive increase in the number of double S100B+/APC-CC1+ and single APC-CC1+ cells. Scale bars: 20 µm (**a**, **b**, **c**); 10 µm (**d**)

**Fig. 9:**
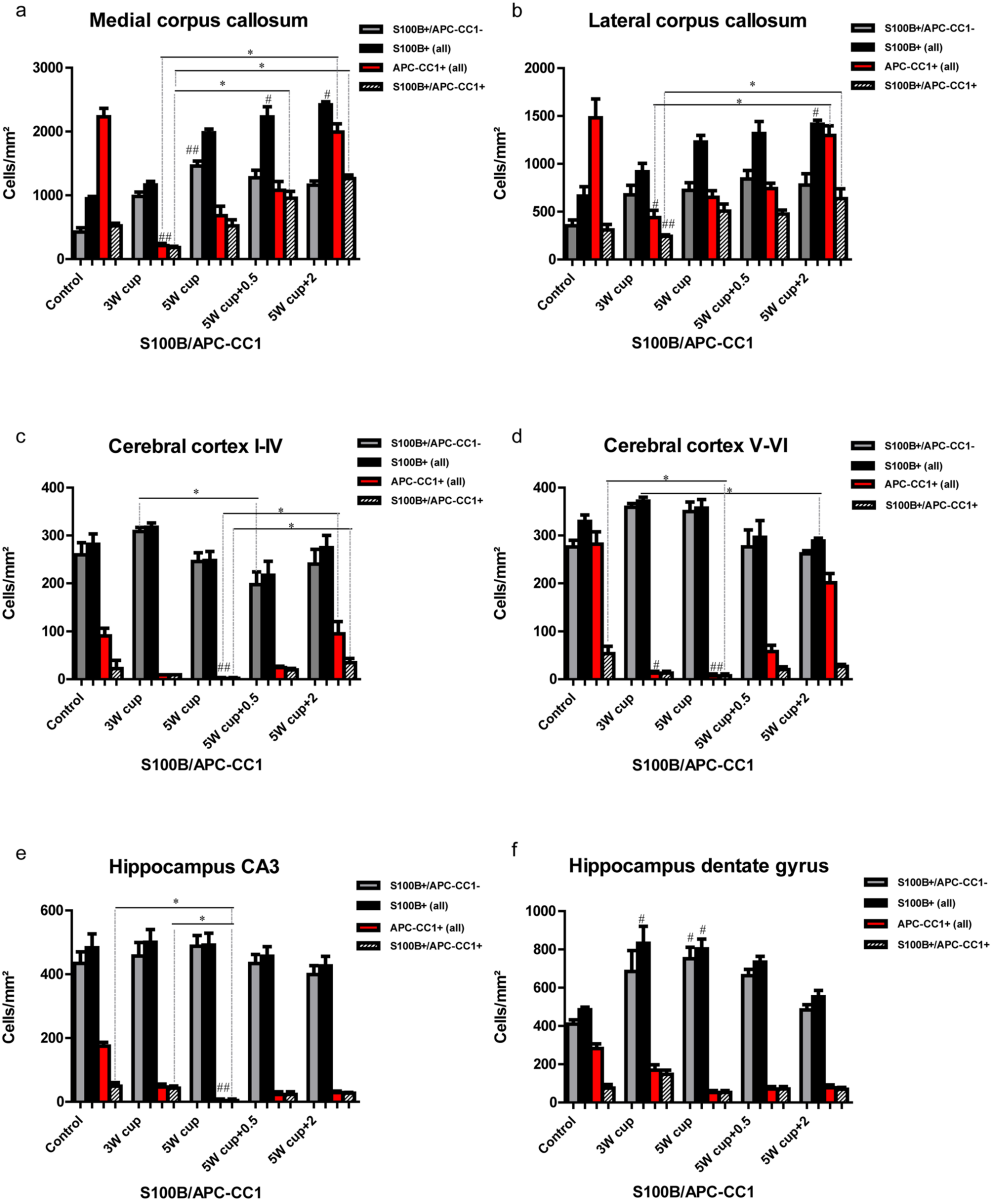
Expression dynamics of S100B and APC-CC1 during the course of de- and remyelination. Graphs represent the qualitative analysis of APC-CC1+ oligodendrocytes (red bars), and S100B+ (black bars) and APC-CC1+/S100B+ cells (striped bars) during the course of de- and remyelination in the different brain regions (3W cup: beginning of demyelination, week 3; 5W cup: severe demyelination, week 5; 5W cup+0.5: early remyelination, week 5.5; 5W cup+2: remyelination, week 7). Graphics **a**–**b** describe the effect of cuprizone on oligodendrocytes (single APC-CC1+ and double S100B+/APC-CC1+ cells) in the medial and lateral part of the corpus callosum. A significant depletion of oligodendrocytes can be observed from the beginning of treatment week 3 (red and striped columns). By contrast, during cuprizone treatment astrocytes (single S100B+ cells) proliferate, reaching a peak at week 5 in the medial part (gray columns). A progressive increase in the number of single S100B+ and S100B+/APC-CC1+ cells can be observed 0.5 and 2 weeks after cuprizone removal, corresponding to the recovery of myelin during remyelination. The graphics **c**–**d** and **e**–**f** show similar expression patterns for the cerebral cortex and hippocampus, respectively. In both areas, oligodendrocytes depletion becomes statistically evident at week 5. No clear patterns of remyelination can be noted in the regions CA3 and dentate gyrus regarding expression dynamics of double S100B+/APC-CC1+ and single APC-CC1+ cells. Bar charts show mean values and SEMs. Significant effects versus controls are indicated by rhombs (*#p* < 0.05,* ##p* < 0.01, and *###p* < 0.001) and significant effects versus different time points are indicated by a line and asterisks (*p < 0.05, **p < 0.01, and ***p < 0.001), *n* = 4.

We hypothesized that GFAP−/S100B+ cells (in the central corpus callosum: 55% in the controls, 24% and 31% during early and late demyelination, respectively, 42% during late remyelination) correspond presumably to oligodendroglia offspring cells. Cuprizone application caused a death/decline of mature APC-CC1-expressing oligodendrocytes in all areas analyzed. OPC proliferated and differentiated into new myelinating oligodendrocytes from week 4 onwards (Skripuletz et al. 2011; Gudi et al. 2014). To provide insight into S100B expression patterns of oligodendrocyte lineages during cuprizone-induced de- and remyelination, we conducted double immunochemical analyses with S100B and APC-CC1, a marker for mature oligodendrocytes. Co-localization of both markers was detected at variable extents in all brain areas analyzed (Figs. 8 and 9). In control animals, all brain regions assessed exhibited only a small population of APC-CC1+ oligodendrocytes concomitantly expressing S100B (23% in the medial corpus callosum; 21% in the lateral corpus callosum; 19% in the cortex; 28% in the CA3, and 27% in the dentate gyrus) (Figs. 8 and 9a–f). During demyelination (week 3–5 of cuprizone feeding), the percentage of the APC-CC1/S100B double-positive cells increases (up to 85% in the medial corpus callosum; up to 78% in the lateral cc; 100% in the cortex; 100% in the CA3 and 100% in the dentate gyrus).

However, an important predominance of single APC-CC1+ oligodendrocytes devoid of S100B expression was again observed in the corpus callosum and the cortex of animals with ongoing remyelination (Figs. 8 and 9). The hippocampus, in contrast, did not obey this pattern during remyelination (Figs. 9e, f). These results confirm and extend previous data, underlining the importance of S100B for recognition of different maturation stages in oligodendroglia, if not the existence of different cell populations (Deloulme et al. 2004).

### Activated microglia do not express S100B during de- and remyelination

**Fig. 10:**
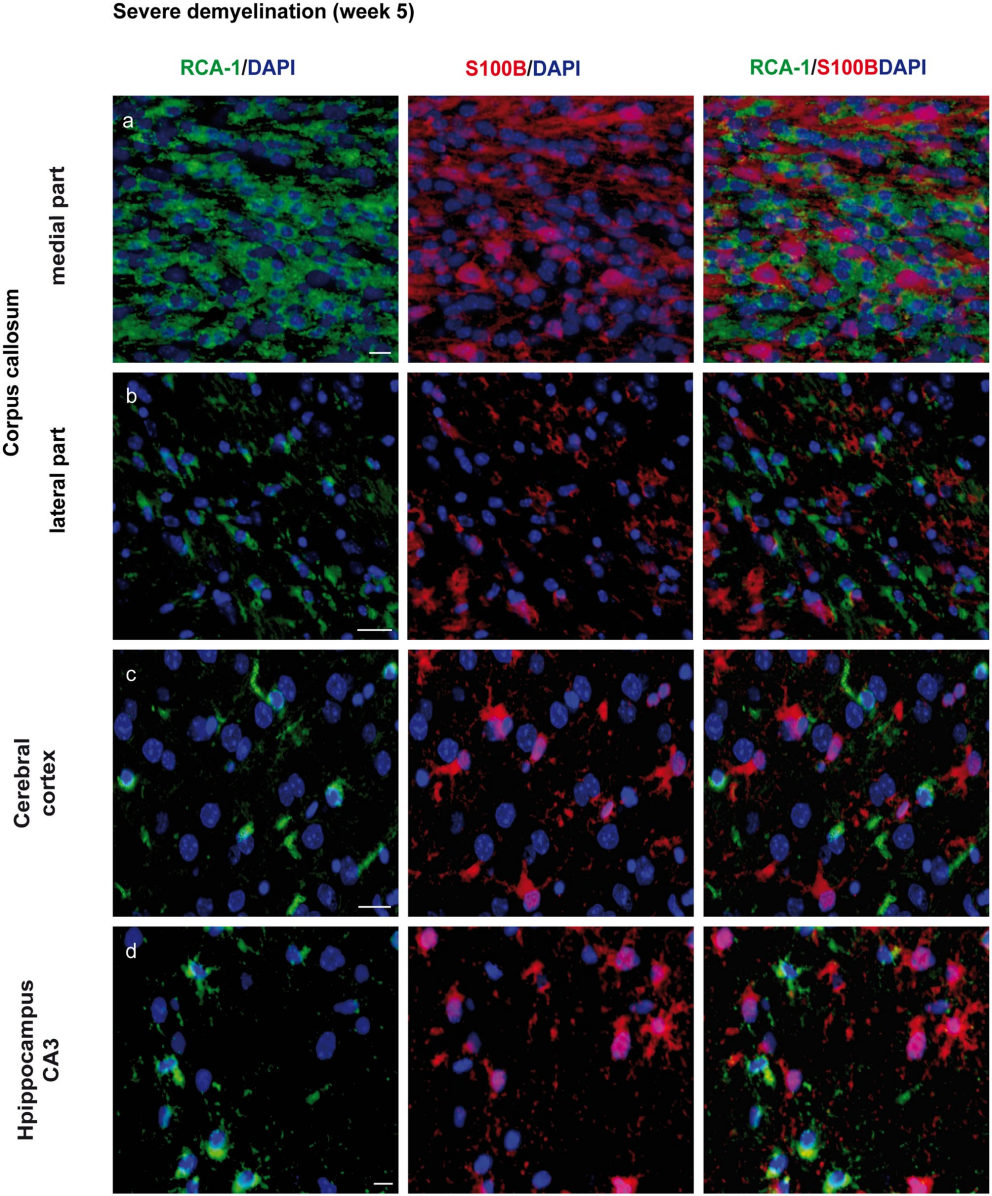
Immunohistochemical analysis of S100B and RCA-1 (marker for activated microglia) expression. Representative pictures demonstrate double immunofluorescence staining with S100B (red) and ricinus communis agglutinin-1 (RCA-1) (green) performed in the central corpus callosum (**a**), cortex (represented layers V–VI in (**b**), and hippocampal areas CA3 (**c**) and dentate gyrus (**d**) of C57BL/6 mice at week 5 under cuprizone diet (severe demyelination). Co-localization of S100B with RCA-1 was not found. Scale bars: 10 µm (**a**, **d**), 20 µm (**b**, **c**)

It is widely known that cuprizone-induced demyelination in the murine CNS is accompanied by strong microglia infiltration/activation (weeks 3–5) (Hiremath et al. 1998; Gudi et al. 2009). Thus, we co-stained the brains of cuprizone-treated mice with S100B and RCA-1 (a marker for activated microglia) and confirmed the absence of S100B expression in microglia in our mouse model (Fig. 10).

### LCN2 is primarily expressed by activated astrocytes in the medial corpus callosum

In this study, we aimed to characterize regional differences in LCN2 expression and its potential as an immunohistochemical marker for reactive gliosis during de- and remyelination.

**Fig. 11:**
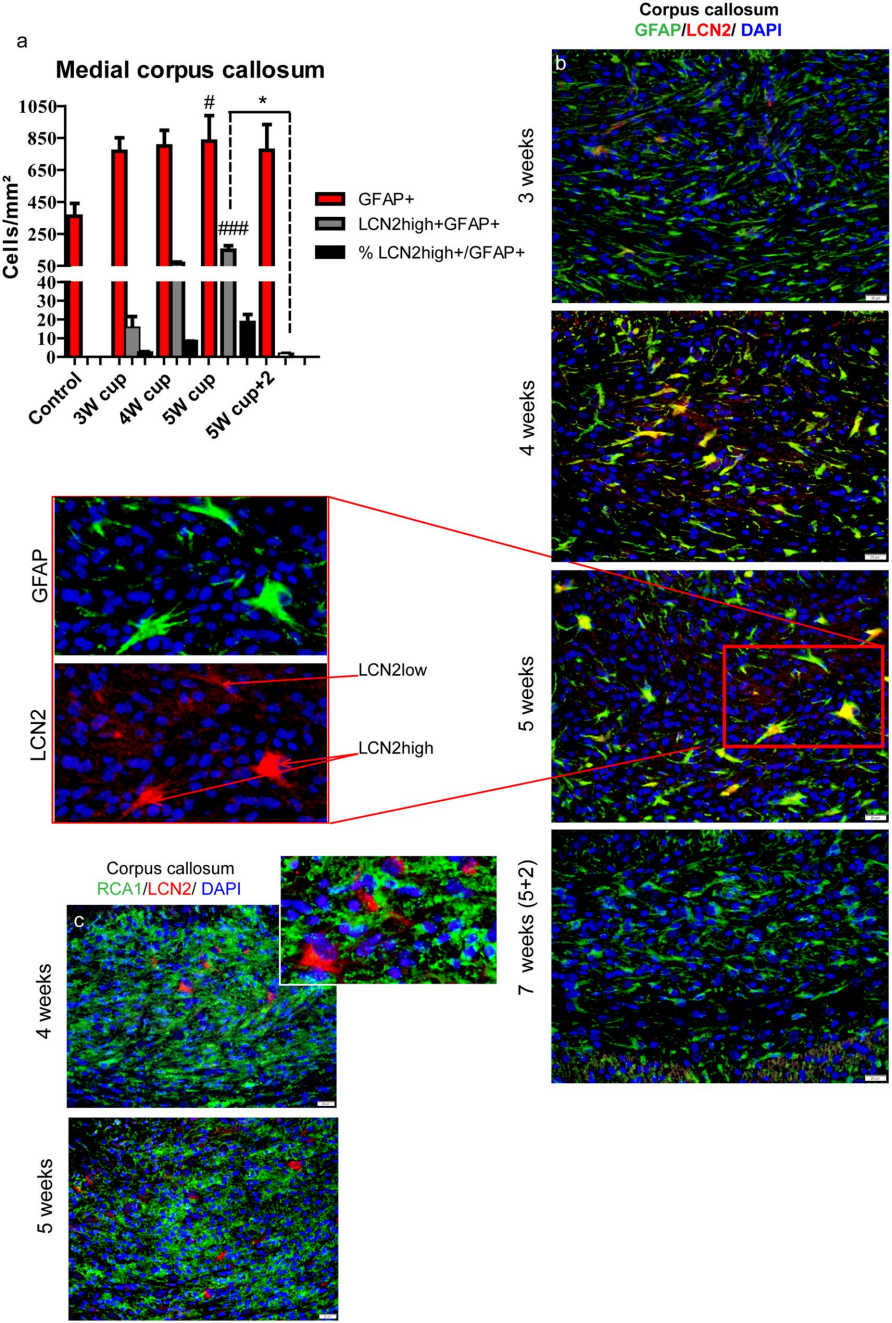
Immunohistochemical analysis of LCN2 expression during cuprizone-induced de- and remyelination in the central corpus callosum. The graph in (**a**) represents a qualitative analysis of GFAP+ astrocytes (red bars) and double GFAP+/LCN2^high^+ astrocytes (gray bars) during the course of de- and remyelination in the central corpus callosum (3W cup: beginning of demyelination, 3 weeks; 5W cup: severe demyelination, 5 weeks; 5W cup+2: remyelination, 7 weeks). Please note that almost all astrocytes in the central corpus callosum displayed very weak, diffuse LCN2 staining at week 5. We could not consider it conclusively as specific staining; thus we count only highly LCN2-positive astrocytes (+LCN2^high^). Black bars represent the percentages of GFAP+/LCN2^high^+ to/from all GFAP+ astrocytes. Bar charts show mean values and SEMs. Significant effects versus controls are indicated by rhombs (*#p* < 0.05,* ##p* < 0.01, and *###p* < 0.001) and significant effects versus different time points are indicated by a line and asterisks (*p < 0.05, **p < 0.01, and ***p < 0.001), *n* = 4–7. Representative pictures (**b**) demonstrate double immunofluorescence staining with LCN2 (red) and GFAP (green) performed in the central corpus callosum during cuprizone-induced de- and remyelination. LCN2 (red) does not co-localize with RCA-1 (green), shown in **c**. Scale bars: 20 µm (**b**, **c**)

In our study, LCN2 staining was associated with GFAP-positive reactive astrocytes but not with activated microglia (Fig. 11). In the medial corpus callosum, LCN2 levels increased during the demyelination at the third week of cuprizone administration, reached its peak at week 5 and rapidly decreased with onset of remyelination (Fig. 11). At that time we could observe weak/diffuse LCN2 staining (LCN2^low^) by nearly all GFAP-positive astrocytes in the corpus callosum; however, only 8% and 18% of all GFAP-positive astrocytes (LCN2^high^) strongly co-express LCN2 at week 4 or week 5, respectively (Fig. 11). In the lateral corpus callosum LCN2 was hardly expressed at all time points studied, whereas in the CA3 region of the hippocampus LCN2 expression was sporadically observed at week 3 and 5 (data not shown). Only few LCN2 positive cells could be detected during acute demyelination in the cerebral cortex near to the corpus callosum.

## Discussion

Due to the involvement of astrocytes in the pathogenesis of degenerative and demyelinating diseases like MS and an increasing awareness of the molecular and functional heterogeneity of these glial cells (Morel et al. 2017), astrocytes are in the focus of numerous current investigations. Recent studies have suggested astrocytes to play a pivotal role in remyelination in animal models and in MS (Moore et al. 2011; Skripuletz et al. 2013; Correale and Farez 2015). Moreover, lines of evidence confirm that astrocytes are a highly heterogenic population (Oberheim et al. 2012). In this context, new astrocytic markers have become an important field of research.

The current work revealed that astrocyte-specific genes *Gfap*, *Vim*, *Aldh1l1*, and *Lcn2* were strongly regulated during the cuprizone-induced de- and remyelination. However, there were clear regional differences concerning their temporal expression pattern as well as their mRNA expression levels, generally between gray and white matter structures. Expression of *Gfap*, *Vim*, and *Aldh1L1* mRNAs reached their maximum in gray matter structures significantly earlier than in the corpus callosum. In contrast, *Lcn2* mRNA expression showed the same temporal pattern in all brain regions studied, but was most upregulated in the corpus callosum.

Immunohistochemical studies revealed that vimentin expression mostly coincided with that of GFAP during the course of de- and remyelination. However, single VIM+ cells were observed in the endothelium of blood vessels and ependymal cells of the gray matter, which corresponds with previous analyses (Schnitzer et al. 1981; Hibbits et al. 2012).

The novel marker ALDH1L1 (Cahoy et al. 2008; Winchenbach et al. 2016) stands out for its high astrocyte specificity and broad expression in healthy and diseased states. Although its exact link in the astrocytic machinery still remains to be elucidated, ALDH1L1 is known to play a crucial role in folate metabolism as a prerequisite for nucleotide biosynthesis and cell division (Krupenko 2009).

The assessment of ALDH1L1 and GFAP via light microscopy revealed a systemic upregulation with complete overlap of both markers during cuprizone-induced demyelination and subsequent remyelination, which may mirror the increased demand for nucleotides as astrocytes proliferate and become activated under inflammatory conditions induced by cuprizone administration (Yang et al. 2011). In accord with earlier reports, we observed ALDH1L1, unlike GFAP, to be useful for astrocyte identification in the cerebral cortex of wild-type animals (Yang et al. 2011; Morel et al. 2019), although fluorescence was significantly weaker here than in fibrous white matter or reactive astrocytes, but similar to that of the hippocampal region. Furthermore, small populations of ALDH1L1+/OLIG2+ cells were detected in the corpus callosum and fimbria adjacent hippocampus only at week 5 of cuprizone treatment, which is consistent with previous findings of our lab wherein GFAP+/OLIG2+ cells (13%) were transiently found in the corpus callosum of cuprizone-challenged mice (Salinas Tejedor et al. 2015). Similar results were previously reported using BAC ALDH1L1-eGFP and ALDH1L1-CreERT2 transgenic mice (Yang et al. 2011). According to current literature, this differentiation of OLIG2-positive progenitors toward astrocytes or oligodendrocytes may occur in a spatiotemporal- and context-dependent manner (Tatsumi et al. 2008; Ono et al. 2009).

S100B has been used to label astrocytes for decades. Still, the wide cellular spectrum and the discovery of more suitable astrocyte markers have led to its waning use in immunohistochemistry over recent years (Adami et al. 2004; Steiner et al. 2007). Both beneficial and detrimental effects of S100B on metabolic functions, cytoskeleton modifications, and cell proliferation and differentiation in the CNS have been identified (Rothermundt et al. 2003; Donato et al. 2013). Additionally, it serves as a biomarker in the cerebrospinal fluid (CSF) and serum of patients with stroke, brain trauma, Alzheimer disease, MS, or viral encephalitis, indicating astrocytic damage or dysfunction of the blood–brain barrier (BBB) (Barateiro et al. 2016; Michetti et al. 2019). Our histological study confirms previous data on astrocytic expression of S100B. Nevertheless, a considerable number of S100B+ cells corresponded to the cells of oligodendrocyte lineage, again consistent with previous studies (Steiner et al. 2007).

Anti-APC (clone CC1, APC-CC1) antibodies are known to be reliable for the detection of mature oligodendrocytes (Bhat et al. 1996) in the rodent brain and have been used in numerous studies (Duncan et al. 2018; Kirby et al. 2019; Valerio-Gomes et al. 2018; Heckers et al. 2017; Gingele et al. 2020). Although raised against adenomatous polyposis coli (APC), this APC-CC1 marker was suggested to reflect cross-reactivity with an unrelated antigen (Brakeman et al. 1999; Lang et al. 2013). Bin and colleagues proposed in their study that these antibodies detect the oligodendroglial Quaking-7 protein (Bin et al. 2016). In fact, in situ hybridization and immunohistochemical studies showed predominantly neuronal expression of APC in the adult human and rodent brain (Brakeman et al. 1999; Senda et al. 1998). However, it was later found that APC is expressed by oligodendrocytes as well, particularly during their differentiation (Lang et al. 2013). Additionally, several studies showed that APC and APC-CC1 were upregulated in astrocytes in various pathological conditions (Leroy et al. 2001; Etienne-Manneville and Hall 2001; Lee et al. 2010).

In our previous study, we examined in detail the expression of APC-CC1 during cuprizone-induced de- and remyelination and confirmed that APC-CC1 reliably detected mature oligodendrocytes (Salinas Tejedor et al. 2015). Double staining with the marker OLIG2 showed that these cells belong to the oligodendroglial lineage. Single positive APC-CC1 cells were not found. In addition, APC-CC1 and the myelin protein CNPase were expressed simultaneously, further confirming that these APC-CC1+ cells were of oligodendroglial origin (Salinas Tejedor et al. 2015). In our previous studies, APC-CC1 did not co-localize with GFAP (Salinas Tejedor et al. 2015). Behrangi and colleagues reported the same pattern of APC-CC1 staining in wild-type mice exposed to cuprizone (Behrangi et al. 2021). However, in the cuprizone-challenged mice expressing eGFP under the human GFAP promoter, a significant proportion of eGFP+ cells were co-labeled with the anti-CC1 antibodies used. The authors argued that because of the perinuclear localization of APC-CC1 and the sometimes one-sided distribution of GFAP in astrocytes, co-localization studies may be complicated. On the other hand, the expression of eGFP (thus also GFAP) in cells of the oligodendrocyte lineage could be an artifact of the human GFAP promoter used (Behrangi et al. 2021).

Strikingly, double staining with S100B and APC-CC1 revealed their co-localization, which notably prevailed over single APC-CC1+ cells at weeks 3 and 5 of cuprizone treatment, coinciding with OPC recruitment during acute demyelination (Skripuletz et al. 2011; Gudi et al. 2014). Meanwhile, single APC-CC1+ cells clearly predominated in control animals and in animals during remyelination. Thus, we suppose an oligodendrocyte subclass which co-expresses S100B and APC-CC1, suggesting an important, if not decisive, involvement of S100B in the differentiation of OPC into myelinating cells (Deloulme et al. 2004; Hachem et al. 2005). The recently published study by Du and colleagues provided multiple lines of molecular and genetic evidence that S100B is expressed in newly differentiated myelinating oligodendrocytes during murine CNS development (Du et al. 2021). As prior works have also indicated the presence of S100B in OPC, further experiments with earlier-lineage oligodendrocyte markers are pivotal to enabling a deeper understanding of its regulatory functions (Deloulme et al. 2004; Hachem et al. 2005).

Apart from this, a constant population of GFAP+/S100B− cells is present in the corpus callosum and subgranular layers of the dentate gyrus. Variances of S100B expression in germinal zone astrocytes have already been described by Raponi and colleagues, hypothesizing a correlation of S100B expression with the loss of stem cell potential during astrocyte maturation (Raponi et al. 2007). In addition, since the largest number of GFAP+/S100B− were found in the lateral corpus callosum, where only partial demyelination and mild microgliosis occurred under the effects of cuprizone, a protective role of these cells in a hostile milieu is possible. Furthermore, S100B surpassed GFAP in labeling cortical astrocytes under normal conditions, with higher fluorescence intensity than ALDH1L1, however with additional oligodendrocyte staining (Steiner et al. 2007).

LCN2 can be produced in different organs and various populations of immune cells, such as macrophages, neutrophils, dendritic cells, and CD4+ and B cells (Floderer et al. 2014; Jung et al. 2017; Jung et al. 2016; Meheus et al. 1993; Chakraborty et al. 2012; Kjeldsen et al. 1993; Nam et al. 2014; Lee et al. 2020). LCN2 has been closely associated with CNS inflammation (Marques et al. 2008, 2012; Ip et al. 2011; Xing et al. 2014; Jha et al. 2015). Elevated levels of LCN2 have been detected in the plasma and CSF of MS patients, and in immune cells in CNS lesions in MS tissue sections (Berard et al. 2012). Furthermore, experimental autoimmune encephalomyelitis (EAE) studies imply that LCN2 is a critical mediator of autoimmune inflammation and disease development in this model of MS (Berard et al. 2012; Nam et al. 2014). Finally, LCN2 has been reported to exert detrimental effects on remyelination (Al Nimer et al. 2016) and to be involved in demyelination as well as gliosis progression in an experimental autoimmune optic neuritis model (EAON) (Chun et al. 2015). LCN2 acts as an autocrine modulator of astrocyte polarization in classical inflammatory conditions, induces upregulation of GFAP and morphological changes characteristic of reactive astrocyte phenotype, and may even trigger self-regulatory apoptosis of activated astrocytes (Lee et al. 2009; Jang et al. 2013b).

In our study we observed strong LCN2 expression in reactive GFAP-positive astrocytes mostly during severe demyelination (week 5) in the central corpus callosum, corresponding to the peak of microglial accumulation and activation. Previously, Xing et al. observed increased phagocytic activity of LCN2-treated microglia (Xing et al. 2014). Lee et al. 2011 showed that cultured neonatal astrocytes express CXCL10 and induce microglial migration in response to stimulation with recombinant LCN2 (Lee et al. 2011). Our own studies revealed that in the cuprizone demyelination model, activated astrocytes produced CXCL10 and attracted microglia to demyelinated lesions (Skripuletz et al. 2013). However, microglia begin to accumulate in the medial corpus callosum of cuprizone-treated mice already at weeks 2–3. Interestingly, at this time, distinct LCN2^high^-positive astrocytes were only sporadically seen in the medial corpus callosum, although upregulation of LCN2 mRNA was observed in this area. It is likely that the expression of LCN2 at week 3 was relatively weak and thus below the detection level of the antibodies used in this study. Generally, only a small population of GFAP-positive astrocytes in the corpus callosum strongly upregulate LCN2. We speculate that in our animal model for demyelination, astrocytes may trigger their activation in an autocrine manner and then modulate phagocytotic activity of microglia as well as their self-regulatory apoptosis via tightly controlled LCN2 level production/secretion. Lee and colleagues proposed that LCN2 can induce deramification and sensitize activated microglia to a self-regulatory apoptosis (Lee et al. 2007). LCN2^high^-positive astrocytes were only sporadically observed in the hippocampus, the cortical layer 6 directly in the vicinity of the corpus callosum, capsula interna, and thalamus at different time points (data not shown), pointing to possible regional peculiarities of astrocytic populations and probably also their functions.

In summary, we found vimentin to be similar to GFAP in staining protoplasmic and fibrous astrocytes. ALDH1L1 was proven/confirmed to be a precise tracer for both fibrous and protoplasmic astrocytes in CNS white and gray matter. We also discovered an ephemeral small population of ALDH1L1+/OLIG2+ cells in the corpus callosum and hippocampus of cuprizone-treated mice. We infer that these cells are pro-astrocytes, capable of de-differentiating into oligodendrocytes under pathological circumstances, or alternatively, that this is a population of stem cells that are capable of differentiating into different cell types. The use of S100B is primarily eligible in the cerebral cortex and should be combined with other astroglial markers, such as ALDH1L1 or a wide-range oligodendroglia marker for the exclusion of oligodendrocytes in the scrutiny of astrocytes. In general, S100B was detected in most GFAP-positive astrocytes, particularly in the cerebral cortex and CA3 region of the hippocampus, throughout the course of cuprizone treatment. However, in the central corpus callosum and dentate gyrus of the hippocampus, some GFAP+ cells lacked S100B.

Numerous GFAP-positive astrocytes in the lateral corpus callosum did not express S100B, indicating the heterogeneity of the astrocyte population.

LCN2 seems to label particular populations of astrocytes temporally, being an interesting candidate to study regional differences in astrocytic populations as an activation marker and thus a potential immunohistochemical marker for reactive gliosis in demyelinating processes.

## Data Availability

The data used in this study are available from the corresponding authors upon reasonable request.
